# Increased expression of cell adhesion molecules in myofasciitis

**DOI:** 10.3389/fneur.2023.1113404

**Published:** 2023-05-09

**Authors:** Xue Ma, Huajie Gao, Li Xu, Zhuajin Bi, Suqiong Ji, Bitao Bu

**Affiliations:** ^1^Department of Neurology, Air Force Medical University Tangdu Hospital, Xi’an, China; ^2^Department of Neurology, Tongji Hospital, Tongji Medical College, Huazhong University of Science and Technology, Wuhan, China

**Keywords:** myofasciitis, dysregulated immune response, cell adhesion molecules, endothelial activation, myopathy

## Abstract

**Background:**

Myofasciitis is a heterogeneous group of diseases pathologically characterized by inflammatory cell infiltration into the fascia. Endothelial activation plays a critical role in the pathogenesis of the inflammatory response. However, the expression of cellular adhesion molecules (CAMs) in myofasciitis has not been investigated.

**Methods:**

Data on clinical features, thigh magnetic resonance imaging, and muscle pathology were collected from five patients with myofasciitis. Immunohistochemical (IHC) staining and Western blot (WB) of the muscle biopsies from patients and healthy controls were performed.

**Results:**

Increased levels of serum pro-inflammatory cytokines, including IL-6, TNF-α, and IL-2R, were detected in four patients. IHC staining and WB indicated significantly increased expression of cell adhesion molecules in blood vessels or inflammatory cells within the perimysium in muscle and fascia tissues of patients with myofasciitis compared to controls.

**Conclusion:**

The up-regulation of CAMs in myofasciitis indicates endothelial activation, which may be potential therapy targets for the treatment of myofasciitis.

## Introduction

Myofasciitis is recognized as a connective disease disorder (CTD) clinically characterized by symmetrical, painful swelling and progressive induration of the extremities and trunk. As a heterogeneous group of diseases, myofasciitis primarily consists of eosinophilic fasciitis (EF) (1), macrophagic myofasciitis (2), immune checkpoint inhibitor-induced myofasciitis (3), infection-associated myofasciitis (4), necrotizing fasciitis (5), chronic graft-versus-host disease-related myofasciitis (6), sarcoid myofasciitis (7), and unknown risk factors-associated myofasciitis. The pathological features of myofasciitis include fascial thickening, marked vasculitis, and inflammatory infiltration by lymphocytes, macrophages, and plasma cells (8).

Cell adhesion molecules (CAMs), including intercellular adhesion molecule 1 (ICAM-1), vascular cell adhesion molecule 1 (VCAM-1), platelet endothelial cell adhesion molecule (PECAM-1/CD31), and CD146, are expressed on vascular endothelial cells and are essential mediators of leukocyte–endothelial cell interactions during the inflammatory response (9, 10). To migrate into the target regions, the circulating leukocytes are activated by inflammatory cytokines and chemokines secreted locally by inflammatory cells, endothelial cells and histocytes, to express integrins on their surface, such as very late antigen 4 (VLA-4, α4/β1, CD49d/CD29) and lymphocyte function-associated antigen 1 (CD11a/CD18), which enable firmer adhesion to other endothelial CAMs (9). The interaction between leukocytes and the endothelium is a major contributor in the inflammatory process (11, 12). However, the role of CAMs in myofasciitis has not been explored. To further understand the physiopathology mechanism of myofasciitis, an analysis of the expression analysis of CAMs may be of vital importance. We investigated the expression of ICAM-1 and its receptor (CD11a/18), VCAM-1 and its receptor (VLA-4), PECAM-1, and CD146 in muscle biopsies from patients with myofasciitis.

## Materials and methods

### Patient selection

Five patients who fulfilled diagnosis criteria for myofasciitis at Department of Neurology, Tongji Hospital were included. Biopsy specimens of muscle and fascia, as well as clinical and laboratory data obtained from these patients were retrospectively analyzed. The diagnosis of myofasciitis was determined based on suggestive clinical and laboratory findings, as well as muscle biopsy or magnetic resonance imaging (MRI) abnormalities [Table 1; (1)]. Other pathological entities, such as scleroderma, autoimmune myopathy, malignancies, and infection were excluded. There was no family history of neuromuscular disorders and no skin rashes were detected in the included patients. No vaccination was performed 1 year before the symptoms occurred. The fascia and muscle biopsy sites were determined by MRI. Muscle and fascia tissue from five patients without neuromuscular disorders served as controls. The study was approved by the Ethics Committee of Tongji Hospital and written informed consent was obtained from all included patients.

**Table 1:** Diagnostic criteria of myofasciitis.

Items
Major criteria:
Myalgia, swelling pain or symmetrical subcutaneous sclerotic induration on limbs
Minor criteria 1:
Fascial thickening with infiltration of lymphocytes and macrophages with or without eosinophils
Minor criteria 2:
Hyperintense fascia on MR fat suppression T2-weighted images
Exclusion criteria: system sclerosis, idiopathic inflammatory myopathy, infection, and malignancy

A diagnosis with myofasciitis will be made if a patient has the major criterion and one or two of the minor criteria.

MR, magnetic resonance.

### Serologic testing

All patients underwent myositis-specific autoantibody (MAA) and myositis-associated antibody (MSA) screening using a commercial semiquantitative line blot assay (Wuhan Kindstar Diagnostics Co., Ltd., Wuhan, China). MSAs and MAAs included anti-Mi2α and β, anti-TIF1γ, anti-MDA5, anti-NXP2, anti-SAE1, anti-Jo1, anti-SRP, anti-HMGCR, anti-PL7, anti-PL12, anti-EJ, anti-OJ, anti-Ku, anti-PM/Scl-100, anti-PM/Scl-75, and anti-Ro52 antibodies. The anti-nuclear, anti-SSA/Ro60, anti-SSB/La, anti-Sm, anti-RNP, anti-mitochondrial, anti-dsDNA antibodies, and rheumatoid factors (RF) were also evaluated. Serum cytokines were assayed by electro chemiluminescence immunoassay, including interleukin-6 (IL-6) (Roche, 05109442190), tumor necrosis factor-α (TNF-α) (SIMENS, LKNF1), interleukin-2 receptor (IL-2R) (SIMENS, LKIP1), interleukin-8 (IL-8) (SIMENS, LK8P1), and interleukin-10 (IL-10) (SIMENS, LKXP1). The assessment of inflammatory indexes was performed, including erythrocyte sedimentation rate (ESR) and C-reactive protein (CRP).

### Histological, enzyme histochemical, and immunohistochemical studies

All biopsy specimens were rapidly cryopreserved in liquid nitrogen, and then stored at −80°C. The frozen sections (7 μm) were stained with hematoxylin and eosin, acid phosphatase, modified Gömöri trichrome, PAS, NADH tetrazolium reductase, cytochrome C oxidase, and ATPase at pH 4.3, 4.5, and 10.4. Eosinophils were detected by Wright-Giemsa staining. The immunohistochemical (IHC) analysis of cells expressing CD3 (1:200, MAC1477, Bio-Rad), CD4 (1:50, ab133616, Abcam), CD8 (1:50, ab93278, Abcam), CD68 (1:50, ab201340, Abcam), VCAM-1 (1:200, 11,444-1-AP, Proteintech), ICAM-1 (1:200, 10,831-1-AP, Proteintech), VLA-4 (1:200, 19,676-1-AP, Proteintech), CD11a/CD18 (1:1000, ab52895, Abcam), CD31/PECAM-1 (1:2000, ab9498, Abcam), and CD146 (1:200, Ab75769, Abcam) were performed using a 3, 3′-diaminobenzidine Detection Kit; the sections were then counterstained with hematoxylin. For immunofluorescence staining, expression of major histocompatibility complex class I (MHC-I) (1:100, ab23755, Abcam) and deposition of membrane attack complex (MAC) (1:50, Sc-58,935, Santa Cruz Biotechnology) on the fascia and sarcolemma were used with Alexa Fluor conjugated secondary antibody (1:400, Invitrogen) for 1 h. Images were obtained using a microscope (Olympus) or Confocal scanning (Olympus).

A modified semiquantitative scale previously defined by Corinna Preusse was used for analysis (2). Each sample was evaluated on 20 high-power fields (HPF; one HPF as 200×). The average number of cells in 6 randomly selected HPFs per sample was calculated and scored as follows: <5 positive cells, almost no staining (−); 5–20 positive cells, sparse staining (+); 21–50 positive cells, scatter staining (++); and > 50 positive cells, abundant staining (+++). The up-regulation of MHC-I on non-necrotic myofibers and MAC deposition on sarcolemma were assessed as follows: no fibers stained (−); 0–10% of fibers stained (+); 11–30% of fibers stained (++); 31–60% of fibers stained (+++); and 61–100% of fibers stained (++++). For the positive staining on endothelium within the perimysium, the quantity and intensity were estimated separately and subjectively evaluated on a scale of -: absent, +: weak, ++: definite, +++: strong staining) (3). All biopsied sections were assessed by two independent experimenters.

### Western blot

Muscle and fascia tissues from controls or patients with myofasciitis were homogenized in cell lysis buffer (Beyotime, China) supplemented with protease inhibitors (MCE, China) and phosphatase inhibitors (Promoter, China). Tissue lysates were centrifuged at 15000 × g for 15 min. The supernatant was collected and protein concentration was determined using the bicinchoninic acid assay. Total protein (20–50 μg) from each sample was resolved on 8% sodium dodecyl sulfate-polyacrylamide gels and transferred to 0.45 μM nitrocellulose filter membranes (Boster, China). The membranes were blocked with 5% skim milk for 1 h at room temperature to prevent non-specific binding of antibodies and were subsequently incubated over night with the primary antibodies. Primary antibodies include those against: glyceraldehyde-3-phosphate dehydrogenase (GAPDH) (1:5000, 60,004-I-Ig, Proteintech), VCAM-1 (1:1000, Ab134047, Abcam), ICAM-1 (1:1000, 10,831-1-AP, Proteintech), VLA-4 (1:1000, ab81280, Abcam), CD11a/CD18 (1:2000, ab52895, Abcam), CD31/PECAM-1 (1:2000, ab9498, Abcam), and CD146 (1:1000, Ab75769, Abcam). Membranes were washed with Tris-buffered saline with 0.05% Tween-20 and incubated for 1 h at room temperature with horseradish peroxidase-labeled anti-rabbit or anti-mouse secondary antibody (1:5000, Cell Signaling Technology). Blots were developed using enhanced chemiluminescence reagents (Servicebio, China) and evaluated using a charged-coupled device camera (Tanon 4,600). Quantification of immunoreactivity was performed by densitometric analysis using Fiji (NIH) software.

### Statistical analysis

The data are presented as mean ± standard deviation (SD). Mann–Whitney U test was used for two groups. The statistical significance of the differences was evaluated using GraphPad Prism (Version 8.0.1). *p* < 0.05 was considered statistically significant.

## Results

### Case presentations

Five patients with myofasciitis were enrolled. The detailed clinical data of patients are presented in Table 2. Basic information of controls is displayed in Supplementary Table S1.

**Table 2:** Clinical finding of patients with myofasciitis.

Gender	Patient 1	Patient 2	Patient 3	Patient 4	Patient 5	M	M	M	M	F
Age (years) at onset	45	28	55	30	32
Time (months), onset to admission	0.8	5	5	6	15
Clinical presentation	Myalgia and fever	Full-circumference swelling and induration of the distal limbs	Hardness of the proximal and distal extremities, and swelling pain	Myalgia and muscle weakness	Myalgia and fever
Fever	Yes	No	No	No	Yes
MRC	5	5	5	5	4
Joint movement disorder	No	Wrist joint	Radio-ulnar joint; wrist joint; hip joint; knee joint	No	No
ESR/CRP	ESR 55 mm/h	ESR 4 mm/h	CRP 14.5 mg/L	ESR 13 mg/L	ESR 46 mm/h
Eosinophilia	No	No	Yes	No	No
CK level at onset (U/L)	32	28	48	3,169	63
Cytokine	IL-6 8.21 pg./mL; TNF-α, IL-8, IL-10, IL-2R normal	IL-6 18.86 pg./mL; TNF-α 17.8 pg./mL; IL-2R 1762 U/mL; IL-8, IL-10 normal	IL-6 24.96 pg./ml; TNF-α 13.0 pg./mL; IL-2R 1,605 U/mL; IL-8, IL-10 normal	IL-6, TNF-α, IL-2R, IL-8, and IL-10 normal	IL-2R 1,042 U/mL; IL-6 19.94 pg./mL; TNF-α 44.3 pg./mL
Anti-nuclear antibody	Negative	Negative	1:100	Negative	1:3200; SS-A
MAAs and MSAs	Negative	Negative	PM/Scl-75	Jo-1, Ro-52	RO-52
Rheumatoid factor	36	Normal	Normal	Normal	27.5 IU/mL (< 20)
Diagnosis	Myofasciitis	Myofasciitis	Eosinophilic fasciitis	Myofasciitis	myofasciitis
Treatment	GC	GC	GC + FK506	GC+ cyclophosphamide	GC
Outcome	Improved	Improved	Improved	Improved	Improved

CK, creatine kinase; CRP, C reactive protein; ESR, erythrocyte sedimentation rate; GC, glucocorticoids; F, female; FK506, tacrolimus; M, male; MAAs, myositis-specific autoantibody; MSAs, myositis-associated antibody; MRC, Medical Research Council; No., number; IL-6, interleukin-6; IL-2R, interleukin-2 receptor; IL-8, interleukin-8; IL-10, interleukin-10; TNF-α, tumor necrosis factor-α;

#### Patient 1

A 45 year-old man was admitted with fever (37.5–39.5°C) and myalgia for 25 days. No muscle weakness and paresthesia were reported. Normal muscle strength was indicated by Medical Research Council (MRC) grading. Muscle atrophy, hypertrophy, and abnormal pathological reflexes were absent. The level of serum creatinine kinase (CK) was normal (normal range: ≤ 190 U/L). An increased level of IL-6 was noted (8.21 pg./mL, normal range: < 5 pg./mL). Serum ESR was 55 mm/h (Normal range: 0–20 mm/h). The patient was negative for MAA and MSA. The level of rheumatoid factors (RF) was increased (36 IU/mL, normal range: < 20 IU/mL). Patient 1 was responsive to glucocorticoid monotherapy and had a favorable outcome.

#### Patient 2

A 28 year-old man presented with increasing induration of the upper limbs and tight feeling of the extremities for 5 months. Limb muscle strength was normal. The patients experienced difficulty in performing wrist extension and wrist flexion. There was no muscle atrophy, hypertrophy, or abnormal pathological reflexes. The serum CK level was normal. The patient exhibited increased levels of IL-6 (18.86 pg./mL), TNF-α (17.8 pg./mL, normal range: < 8.1 pg./mL), and IL-2R (1762 U/mL, normal range: 223–710 U/mL). MAA and MSA were negative. The patient was responsive to glucocorticoid monotherapy.

#### Patient 3

A 55-year-old man had experienced skin induration involving both forearms and legs with joint mobility disorder for 5 months. Physical examination revealed that the patient had difficulty performing the wrist flexion/extension task, forearm pronation/supination, hip flexion, and knee flexion. Limb muscle strength was normal. Blood eosinophilia (defined as ≥0.5 × 10^9^/mL or 7% of total leukocytes) was evident. The patient was positive for anti-PM/Scl-75 antibodies. The level of serum CK was normal. CRP was elevated (14.5 mg/L, normal range: < 1 mg/L). Increased levels of IL-6 (24.96 pg./mL), TNF-α (13.0 pg./mL), and IL-2R (1,605 U/mL) were observed. The patient fulfilled the diagnostic criteria for EF (1). However, the possibility of CTD-related myofasciitis cannot be entirely excluded. Patient 3 was initially treated with glucocorticoid monotherapy but experienced no improvement. Addition of tacrolimus enabled achievement of clinical remission. The disorder of radioulnar and wrist joint function at the onset and recovery of joint movement are, respectively, displayed in Additional File 1 and 2.

#### Patient 4

A 30-year-old man was admitted with myalgia for 5 months. No joint movement disorder or limb muscle weakness was detected. Serum CK at onset was 3,169 U/L. The patient was positive for anti-Jo-1 and Ro-52 antibodies. The level of ESR was normal. The patient was diagnosed as CTD-related myofasciitis. The patient was administrated glucocorticoid and cyclophosphamide therapies and the serum CK declined to 214 U/L after 6 months.

#### Patient 5

A 32-year-old woman presented with myalgia and fever (37.5–39°C) for 12 months. The patient exhibited decreased limb muscle strength (4/5 MCR grade). No joint movement disorder was observed. The patient had been diagnosed with primary Sjogren’s syndrome by rheumatologists 1 year previously. The serum CK level was normal and the serum anti-nuclear antibody titer was 1:3200. The patient was positive for Ro-52/SSA autoantibodies. The ESR was elevated (46 mm/h). The cytokine test revealed elevated levels of IL-2R, IL-6, and TNF-α (1,042 U/mL, 19.94 pg./mL, and 44.3 pg./mL, respectively). Rheumatoid factor was 27.5 IU/mL. The patient was administered glucocorticoid therapy and the clinical symptoms quickly resolved.

### Upper and lower extremities MRI

**Figure 1:**
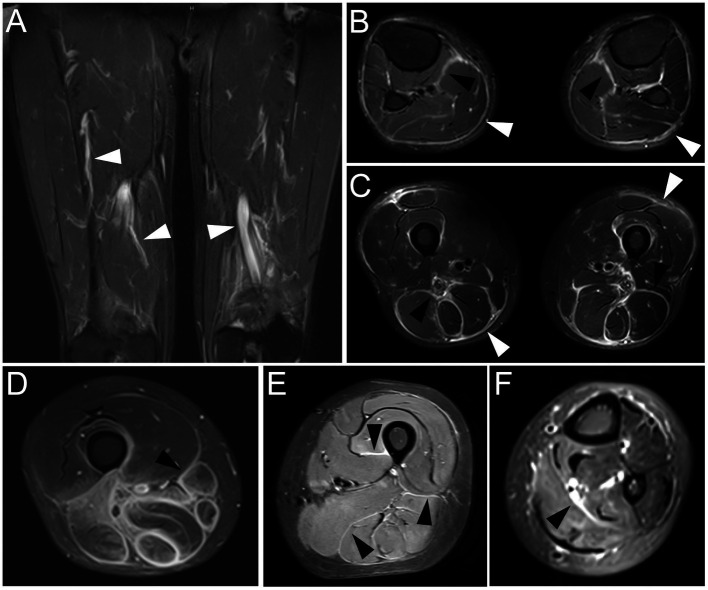
MRI findings at time of diagnosis in five patients with myofasciitis **(A)** Increased signal abnormalities were noted in deep fascia of bilateral fascia of vastus medialis muscle and semimembranosus (white arrowheads) on fat suppression T2-weighted thigh MRI in Patient 1. **(B)** MRI of the lower extremities displayed hyperintensity in superficial fasciae (bright arrowheads) and deep fasciae (black arrowheads) on fat suppression T2-weighted images in Patient 2. **(C)** Fat suppression T2-weighted thigh MRI in Patient 3 showed thickening and increased signal in superficial (bright arrowheads) and deep (black arrowheads) facias of posterior thigh muscles. **(D)** Muscular edema and thickening fascia (black arrowhead) of posterior thigh muscles on fat suppression T2-weighted MRI images were obvious in Patient 4. **(E)** Thickening deep fascia (black arrowheads) had high signal intensity on fat suppression T2-weighted MRI of thigh in Patient 5. **(F)** MRI of forearm in Patient 5 showed hyperintensity in deep fasciae (black arrowhead).

Limbs MRI on fat suppression T2-weighted images indicated striking abnormalities. Thigh images of Patient 1 indicated the hyperintense signal of bilateral fascia of vastus medialis muscle and semimembranosus (Figure 1A). Patchy hyperintensity was distributed in upper arm muscle groups of Patient 1. Thickness and edema of subcutaneous superficial and deep muscular fasciae on lower extremity MRI images of Patient 2 were evident (Figure 1B). Upper arms images of Patient 2 showed slight signal hyperintensity in subcutaneous fatty and perifascicular tissues. Patient 3 demonstrated that marked hyperintense signals in a perimysial and perifascicular distribution in all thigh, calf (Figure 1C), and upper limb muscle groups. Muscular edema and thickening of the fascia of the posterior thigh muscles on fat suppression T2-weighted MRI images were observed in Patient 4 (Figure 1D). Thigh (Figure 1E) and forearm images (Figure 1F) of Patient 5 showed high signal intensity of the thickened deep and superficial fascia. These manifestations were in consistent with myofasciitis in these patients.

### Pathological findings

**Figure 2:**
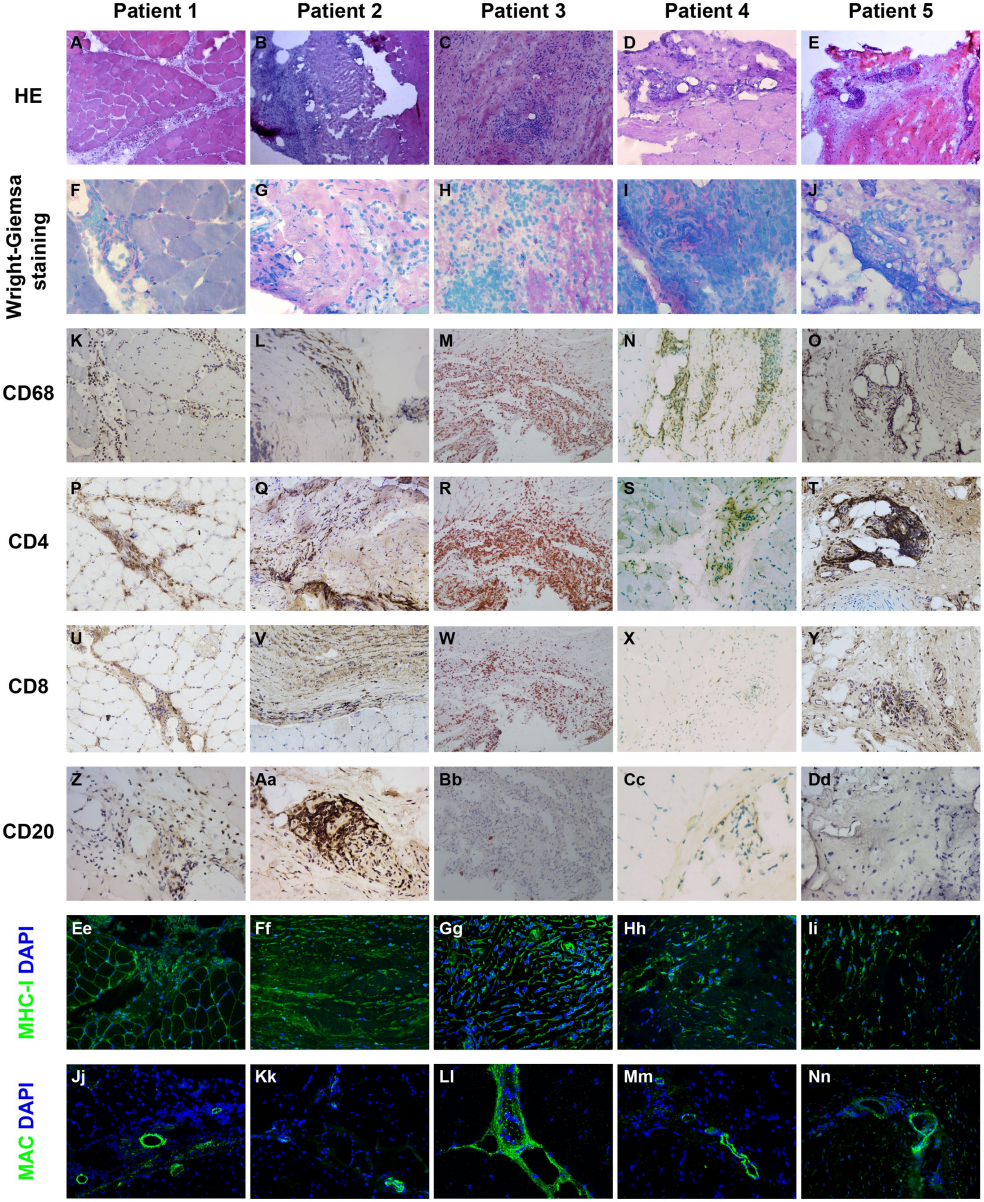
Immunohistochemical illustration of inflammatory infiltrate and immune-mediated factors in biopsied specimen of patients with myofasciitis. **(A–E)** Hematoxylin and eosin staining showed the numerous inflammatory cells in fascia and perivascular collections of inflammatory cells with minimal muscle damage in these patients. Original magnification: ×200. **(F–J)** A few eosinophils (black arrows) were observed in Patient 3. Original magnification: ×400. **(K–O)** A substantial infiltration of CD68^+^ macrophages were noted in the affected fascia in these patients with myofasciitis. Original magnification: ×200. **(P–T)** Abundant CD4^+^ T lymphocytes infiltrated into the fascia in these patients. Scale bar, 200 μm. Original magnification: ×200. **(U–Y)** Scatter CD8^+^ T lymphocytes were found in these patients. Original magnification: ×200. **(Z–Dd)** Scatter staining of CD20^+^ B lymphocytes was noted in fascia of Patient 2. Rare B lymphocytes were detected in Patient 1, Patient 3, Patient 4, and Patient 5. Original magnification: ×400. **(Ee–Ii)** The MHC-I on the sarcolemma close to thickened fascia and inflammatory cells was up-regulated in these patients. Original magnification: ×400. **(Ji–Nn)** MAC was positive with a vascular strong staining pattern in these patients. Original magnification: ×400.

Immunophenotype of inflammatory infiltration, up-regulation of MHC-1, and MAC deposition in biopsied specimens of patients with myofasciitis are presented in Figure 2, Table 3. Numerous inflammatory cells in the thickened fascia and perivascular inflammation were evident in biopsied specimens of patients with myofasciitis (Figures 2A-E). A few eosinophils were detected in the fascial tissues of Patient 3 but not of other four patients (Figures 2F-J). Macrophages and T lymphocytes constituted the predominant mononuclear cellular infiltrate and were dominantly distributed within the collagen tissues (Figures 2K-Y). Scatter B lymphocytes were detected in samples of all five patients (Figures 2Z-Dd). The expression of MHC-1 on the fibroblasts was particularly strong in five patients (Figures 2Ee-Ii). The intensity of MHC-I on the sarcolemma close to the immune cells was up-regulated in Patient 1 and 3. MAC was deposited on blood vessels in the fascia of the five patients (Figures 2Jj-Nn). No positive staining of CD4, CD8, CD68, and CD20 was observed in sections from controls (Supplementary Figure S1). Staining for MHC-I and MAC was detected on blood vessels in fascial tissues of control samples (Supplementary Figure S1).

**Table 3:** Immunophenotype of inflammatory infiltration, up-regulation of MHC-1, and MAC deposition in patients with myofasciitis.

	Patient 1	Patient 2	Patient 3	Patient 4	Patient 5
CD68^+^ macrophages	++	+	+++	+++	++
CD3^+^ T lymphocytes	+++	+++	+++	+++	++
M/T	<1	<1	≈1	≈1	≈1
CD4^+^ T lymphocytes	++	++	+++	++	+
CD8^+^ T lymphocytes	−	+	++	++	+
CD4/CD8	>1	>1	>1	≈1	≈1
CD20^+^ B lymphocytes	+	++	+	+	−
Eosinophils	−	−	+	−	−
Upregulation of MHC-1	+ (myofibers); ++ (blood vessels); ++ (fibroblasts)	- (myofibers); +++ (blood vessels); ++ (fibroblasts)	+ (myofibers); +++ (blood vessels); +++ (fibroblasts)	- (myofibers); ++++ (blood vessels); +++ (fibroblasts)	+ (myofibers); ++ (blood vessels); + (fibroblasts)
MAC deposition	+ (myofibers); +++ (blood vessels)	- (myofibers); +++ (blood vessels)	- (myofibers); ++ (blood vessels)	- (myofibers); +++ (blood vessels)	- (myofibers); +++ (blood vessels)

CD4/CD8, CD4^+^/CD8^+^ T lymphocyte ratio; M/T, macrophage/CD3^+^ T lymphocyte ratio; MHC-I, major histocompatibility complex class I; MAC, membrane attack complex.

**Figure 3:**
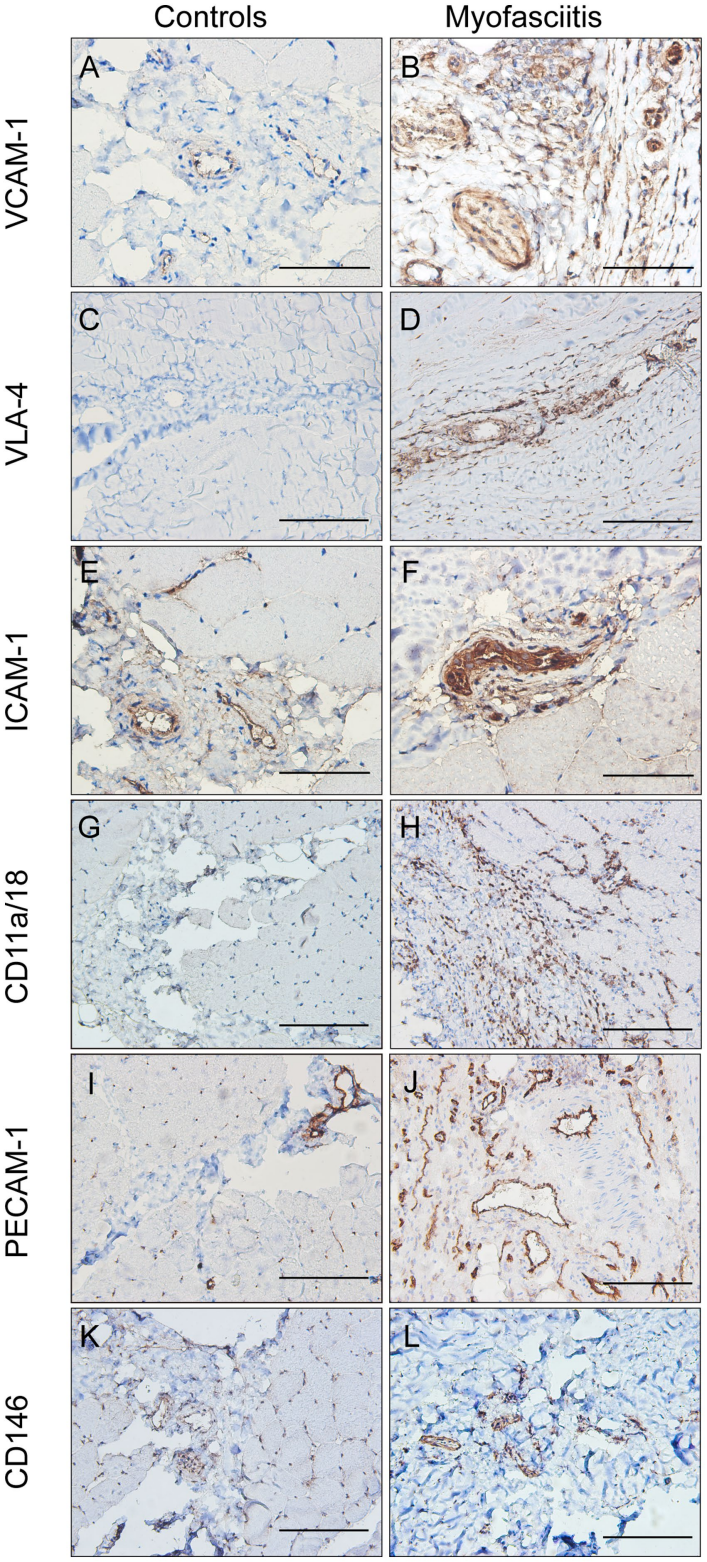
The expression of cell adhesion molecules in controls and patients with myofasciitis. **(A,B)** The weak expression of VCAM-1 in controls and up-regulated VCAM-1 on blood vessels and a few inflammatory cells in Patient 3. A and B, scale bar, 50 μm. **(C,D)** No positive staining of VLA-4 in controls and the expression of VLA-4 on scattered inflammatory cells in Patient 2. C and D, scale bar, 100 μm. **(E,F)** The low-level expression of ICAM-1 on vascular endothelium cells in controls and overexpressed ICAM-1 on vascular endothelial cells and a few inflammatory cells in Patient 2. E and F, scale bar, 50 μm. **(G,H)** No positive staining of CD11a/CD18 in controls and the expression of CD11a/CD18 on scatter inflammatory cells in Patient 3. G and H, scale bar, 100 μm. **(I,J)** The expression of PECAM-1 on blood vessels in controls and up-regulated PECAM-1 on blood vessels in Patient 3, especially in actively involved areas. **(I**,**G)**, scale bar, 100 μm. (**K,L)** Very weak positive staining of CD146 on vascular endothelium cells in controls and strong intensity of CD146 on blood vessels in Patient 2. **(K,L)**, scale bar, 100 μm.

**Figure 4:**
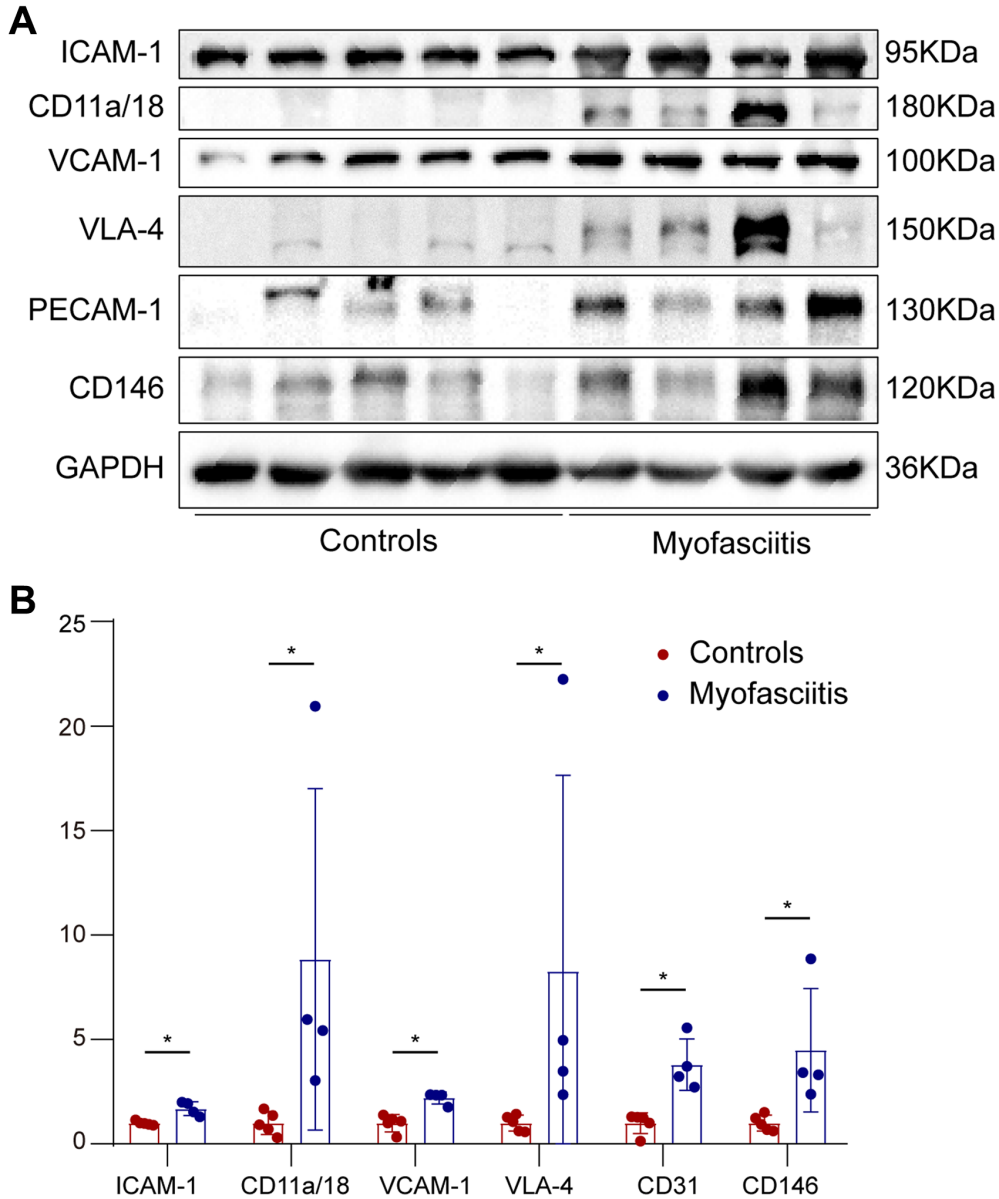
Western blot of cells adhesion molecules in muscle biopsies from controls and patients with myofasciitis. **(A)** Representative Western blot of ICAM-1, CD11a/CD18, VCAM-1, VLA-4, PECAM-1, and CD146 in muscle biopsies for five controls and four patients, including Patient 1, Patient 2, Patient 3, and Patient 5. **(B)** Quantitative analysis of ICAM-1, CD11a/CD18, VCAM-1, VLA-4, PECAM-1, and CD146 relative expression. *n* = 5 for controls, *n* = 4 for myofasciitis. ^*^p < 0.05. Statistical analyses were performed by Mann–Whitney U test.

The expression of CAMs in patients with myofasciitis is summarized in Table 4. Weak positive staining of VCAM-1 in the controls was noted (Figure 3A). However, there was the strong expression of VCAM-1 on vascular endothelial cells within the connective tissue and a few inflammatory cells in patients with myofasciitis (Figure 3B). No positive staining of VLA-4 was observed in the controls (Figure 3C), but there was positive staining of VLA-4 on scatter inflammatory cells in patients with myofasciitis (Figure 3D). There was low-level expression of ICAM-1 on the tissues of controls (Figure 3E) but this was up-regulated in myofasciitis (Figure 3F). CD11a/CD18 was positive on inflammatory cells (Figure 3H), whereas no positive signal was observed in controls (Figure 3G). PECAM-1 was expressed on the vascular endothelium in both controls and myofasciitis (Figures 3I,J). Light expression of CD146 on blood vessels within connective tissue in controls (Figure 3K), whereas CD146 was strongly positive in myofasciitis (Figure 3L). Western blot results were consistent with those of the immunostaining study, and showed that ICAM-1, CD11a/18, VCAM-1, VLA-4, CD31, and CD146 at the protein levels in myofasciitis were significantly increased (Figures 4A,B).

**Table 4:** Intensity of immunohistochemistry staining of cell adhesion molecules and their receptors expression in myofasciitis and HC.

		Patient 1	Patient 2	Patient 3	Patient 4	Patient 5	HC 1	HC 2	HC 3	HC 4	HC 5
VCAM-1	Vascular endothelium	+++	+++	+++	+++	+++	+	+	+	+	++
	Inflammatory cells	+	++	+++	+++	++	−	−	−	−	−
VLA-4	Vascular endothelium	−	−	−	−	−	−	−	−	−	−
	Inflammatory cells	++	++	+++	++	++	−	−	−	−	−
ICAM-1	Vascular endothelium	+++	+++	+++	+++	+++	++	++	++	++	++
	Inflammatory cells	++	++	+	++	+++	−	−	−	−	−
CD11a/CD18	Vascular endothelium	−	−	−	−	−	−	−	−	−	−
	Inflammatory cells	++	++	+++	++	+	−	−	−	−	−
PECAM-1	Vascular endothelium	+++	+++	+++	+++	+++	+++	+++	+++	+++	+++
	Inflammatory cells	+	+	++	+	+	−	−	−	−	−
CD146	Vascular endothelium	++	++	++	+	+	+	+	+	−	+
	Inflammatory cells	+	++	+++	++	++	−	−	−	−	−

VCAM-1, vascular cell adhesion molecule 1; VLA-4, integrin alpha 4/CD49D; ICAM-1, intercellular adhesion molecule 1; PECAM-1, platelet-endothelial cell adhesion molecule

## Discussion

We identified immunopathological changes and the expression of cells adhesion molecules in five patients with myofasciitis, including one patient with EF, two patients with CTD-related myofasciitis, and two patients with unknown triggers, indicating the dysregulated immune response and the elevated expression of CAMs in myofasciitis.

The relationship between eosinophils and myofasciitis remained unclear. A previous study demonstrated elevated levels of serum eosinophilic cationic protein in EF (4). An increased capacity for eosinophilic migration in EF did not correlate with disease activity (5), suggesting that eosinophils may not be involved in the mechanism underlying the onset of this condition. Anti-PM/Scl-IgG-positive patients frequently exhibited extensive extramuscular features, pathologically manifested by perivascular inflammation (6). Perivasculitis was detected in the biopsied specimen of Patient 3 with anti-PM/Scl-75 antibodies. Therefore, the etiology of Patient 3 with EF may be associated with anti-PM/Scl-75 antibodies. Perivasculitis was also identified in other patients, which could be a trigger (1). Patient 4 was positive for anti-Jo-1 autoantibodies. Anti-Jo-1 autoantibodies are the first antibodies to aminoacyl histidyl transfer RNA synthetases to be detected in 15–25% patients with idiopathic inflammatory myopathy (7). Patients with anti-Jo-1 antibodies had unique histological features characterized by perimysial connective tissue fragmentation and inflammation (8). It was previously reported that a patient with positive anti-Jo-1 autoantibodies exhibited pulmonary vasculitis (9). Myofasciitis in Patient 4 may be associated with an anti-Jo-1-mediated disorder of the connective tissue. Patient 5 has been diagnosed with Sjögren syndrome, which may be related to myofasciitis (1). Although the risk factors of Patient 1 and 2 remain unknown, it is important to investigate them.

The up-regulation of CAMs has been demonstrated in inflammatory or autoimmune diseases, such as idiopathic inflammatory myopathies (10–12), multiple sclerosis (13), asthma (14), rhinitis (15), and inflammatory bowel disease (16, 17). Previous research indicated that the CAMs on endothelial cells can be induced by several triggers, including pro-inflammatory cytokines, autoantibodies (18), lipopolysaccharide (19), proteinase 3 (20), reactive oxygen species, and antioxidants (21). Previous study reported increased serum IL-5 (4), hypergammaglobulinemia (22), elevated serum superoxide dismutase levels (23), and circulating overexpression of CD40 ligands (24), were detected in a few patients with EF. Elevated secretion of interferon-γ, IL-2, and leukemia inhibitory factor by peripheral blood mononuclear cells was identified in four cases with EF (25). In this study, elevated serum cytokines in patients with myofasciitis was observed. Therefore, it is reasonable to presume that these inflammatory mediators may be responsible for the endothelial activation. It is supposed that receptors on leukocytes, such as VLA-4 binding to VCAM-1 and CD11a/CD18 binding to ICAM-1 on the surface of activated endothelium cells, may weaken gap formation or junctional endothelial cell–cell interactions that facilitate leukocyte transendothelial migration into the fascia in the pathological process of perivasculitis in myofasciitis.

Blockade of ICAM-1 was a well-established and highly effective treatment in vasculitis (26). The use of monoclonal antibodies and pharmacotherapy against these CAMs reduced the expression of CAMs and ameliorated vasospasm in animal experiment studies of treatment of vasospasm after subarachnoid hemorrhage (27). Therefore, endothelial activation in myofasciitis may be a potential treatment target for myofasciitis.

The limited sample size and the retrospective design from a single medical center were the main limitations of the study. Therefore, paucity of information and selection bias might exist. Studies with larger cohorts from multiple medical centers will further clarify the clinicopathological features of patients with myofasciitis. Another limitation is that all patients were Chinese; our results may therefore not be generalizable to other races.

## Conclusion

To explore the precise therapeutic approaches of myofasciitis, it is of utmost importance to identify the underlying pathogenic mechanisms. The endothelial activation in myofasciitis could potentially be therapeutic targets.

## Data availability statement

The original contributions presented in the study are included in the article/Supplementary material, further inquiries can be directed to the corresponding author.

## Ethics statement

The studies involving human participants were reviewed and approved by the Ethics Committee of Tongji Hospital. The patients/participants provided their written informed consent to participate in this study. Written informed consent was obtained from the individuals, for the publication of any potentially identifiable images or data included in this article.

## Author contributions

BB and SJ contributed to the conceptualization of the study and funding acquisition. XM, HG, ZB, and LX performed the histological staining, western blot and statistical analysis. XM was a major contributor in writing the manuscript. BB has full access to all the data in the study and takes responsibility for the integrity of the data and the accuracy of the data analysis, contributed to critical revision of the manuscript for important intellectual content, and contributed to the supervision. All authors contributed to the article and approved the submitted version.

## Funding

This study was supported by the National Natural Science Foundation of China (Grant number: 81873758 to BB).

## Conflict of interest

The authors declare that the research was conducted in the absence of any commercial or financial relationships that could be made as a potential conflict of interest.

## Publisher’s note

All claims expressed in this article are solely those of the authors and do not necessarily represent those of their affiliated organizations, or those of the publisher, the editors and the reviewers. Any product that may be evaluated in this article, or claim that may be made by its manufacturer, is not guaranteed or endorsed by the publisher.
