# Supplementary material for: Increased expression of cell adhesion molecules in myofasciitis
Source: Front Neurol. 2023 May 9;14:1113404. doi: 10.3389/fneur.2023.1113404 (PMC10203699; doi:10.3389/fneur.2023.1113404)
Supplement: Supplementary file 1 [file Table_1.DOCX]

Supplementary Material

**Increased expression of cell adhesion molecules in myofasciitis**

**Xue Ma^1, 2^; Huajie Gao^2^; Li Xu, MD^2^; Zhuajin Bi^2^; Suqiong Ji, MD ^2^;** **Bitao Bu, MD, PhD^2^ ***

1 Department of Neurology, Air Force Medical University Tangdu Hospital, Xi’an, China

2 Department of Neurology, Tongji Hospital, Tongji Medical College, Huazhong University of Science and Technology, Wuhan, China

* Correspondence:
Bitao Bu

No.1095, Jiefang Avenue, Qiaokou District, Wuhan, China

[bubitao@tjh.tjmu.edu.cn](mailto:bubitao@tjh.tjmu.edu.cn)

# Supplementary Figure and Table

## Supplementary Figure

**Supplementary Figure 1**


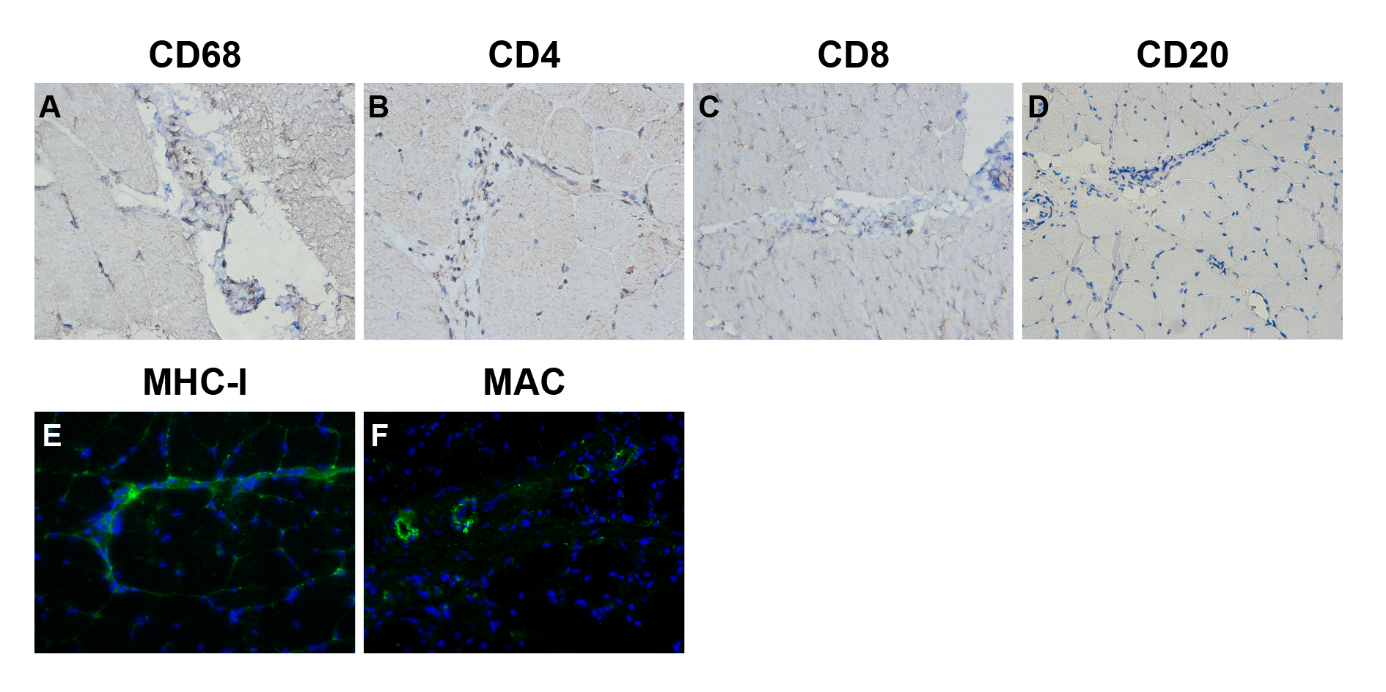


**Supplementary Figure 1.** **The staining pattern of inflammatory infiltrate, MHC-I, and MAC in biopsied specimen of controls**

(A-D) No positive staining of CD68^+^ macrophages, CD4^+^ T lymphocytes, CD8^+^ T lymphocytes, and CD20^+^ B lymphocytes in fascia from controls. Original magnification: ×400.

(E-F) A weak staining pattern of MHC-I and MAC on vessels in fascia from controls. Original magnification: ×400.

**1.2 Supplementary Table 1. Basic information from Controls**

| Items | Controls |
| --- | --- |
| N | 5 |
| Age | 40 (36-55) |
| Female | 1 |
| Muscle weakness | 0 |
| Myalgia | 0 |
| MRC at biopsy | 5 |

Abbreviations: N, number; MRC, Medical Research Council
